# The *AP2*-Like Gene *OitaAP2* Is Alternatively Spliced and Differentially Expressed in Inflorescence and Vegetative Tissues of the Orchid *Orchis italica*

**DOI:** 10.1371/journal.pone.0077454

**Published:** 2013-10-21

**Authors:** Marinella Salemme, Maria Sica, Giovanni Iazzetti, Luciano Gaudio, Serena Aceto

**Affiliations:** Department of Biology, University of Naples Federico II, Napoli, Italy; University College London, United Kingdom

## Abstract

The AP2/ERF proteins are plant-specific transcription factors involved in multiple regulatory pathways, from plant organ development to response to various environmental stresses. One of the mechanisms that regulates the *AP2-like* genes involves the microRNA miR172, which controls their activity at the post-transcriptional level. Extensive studies on *AP2-like* genes are available in many different species; however, in orchids, one of the largest plant families, studies are restricted to a few species, all belonging to the Epidendroideae subfamily. In the present study, we report the isolation of an *AP2-like* gene in the Mediterranean orchid *Orchis italica* (Orchidoideae). The *OitaAP2* locus includes 10 exons and 9 introns, and its transcript is alternatively spliced, resulting in the long *OitaAP2* and the short *OitaAP2_ISO* isoforms, with the latter skipping exon 9. Both isoforms contain the conserved target site for miR172, whose action is demonstrated by the presence of cleaved *OitaAP2* mRNA. The *OitaAP2* and *OitaAP2_ISO* mRNAs are present in the tepals and lip before and after anthesis at different expression levels. In addition, the *OitaAP2_ISO* isoform is expressed in the ovary before pollination and in the root and stem. The isoform-specific expression pattern suggests a functional differentiation of the *OitaAP2* alternatively spliced transcripts. The expression profile of miR172 is complementary to that of the *OitaAP2* isoforms in inflorescence tissues before anthesis, whereas after anthesis and in ovary tissue before and after pollination, this relationship disappears, suggesting the existence of *OitaAP2* inhibitory mechanisms in these tissues that differ from that involving miR172.

## Introduction

The large plant-specific superfamily APETALA2/Ethylene-Responsive element-binding Factor (AP2/ERF) includes transcription factors involved in various regulatory pathways. The highly conserved DNA-binding domain AP2 is shared by all members of the superfamily [1], [2]. Based on the number of AP2 domains and the presence of other functional domains, the superfamily is divided into three families: AP2-like/AINTEGUMENTA (ANT), ERF-like and RAV. The AP2-like/ANT family, in which two AP2 domains are present [3], includes proteins that act in multiple stages and tissues during plant development [4], [5], [6], [7]. Sequence comparison revealed that the AP2-like group can be further divided into AP2 and the RELATED TO AP2 (RAP2) groups [2]. The ERF-like family and the RAV family are both characterized by a single AP2 domain, and their members are involved in the response to various environmental stresses [8], [9], [10], [11]. In addition to the single AP2 domain, the RAV family contains the DNA-binding domain B3 [12].

The AP2-like proteins (both AP2 and RAP2) are encoded by mRNAs containing a conserved target site for the microRNA miR172 that regulates their activity at the post-transcriptional level, predominantly through mRNA cleavage and translational inhibition [13], [14], [15]. To date, a number of studies have analyzed the *AP2-*like genes; the majority of studies have focused on dicots and occasionally monocots (mainly Poaceae) [16], [17], [18], gymnosperms [19], [20], [21] and ferns [3].

The *AP2*-like genes are involved in reproductive phase transition and flower development. Within the ABCDE model of flower development [22], [23], [24], [25], the *AP2-*like genes belong to the A-class and, alone or together with the B-class genes, are mainly involved in the specification of the identity of outer floral whorls (sepals and petals) [4], [22], [26], [27], [28], in ovule, seed and post-embryonic development [4], [29], [30], [31], [32]. The A- and C-class functions are mutually exclusive, even though recent studies conducted in *Arabidopsis* demonstrated that the balance in expression of these two gene classes, moreso than the presence/absence of their gene products, regulates the correct formation of outer and inner floral whorls [27]. AP2-like proteins interact with their specific binding sites located within intron 2 of the C-class *AG* gene, repressing its expression [33]. In addition to their role in flower development, some *AP2*-like genes are also expressed in vegetative tissues [2], [34], [35].

The analysis of genes involved in the flower development pathway in model species is of great relevance (e.g., *Arabidopsis* and *Oryza*) to the understanding of the general mechanisms that govern the formation of the floral organs. However, as revealed by the extension of the study of the B- and C-class genes to non-model species (such as orchids), gene functions often do not overlap, completely or in part, between these species and model species [36], [37], [38], [39].

The family Orchidaceae is one of the largest among the flowering plants and is characterized by highly diversified flowers and reproductive strategies. The orchid flower is zygomorphic, with three outer tepals, two inner lateral tepals and an inner median tepal called labellum or lip. The male and female reproductive tissues are fused into a single structure known as column, at the base of which the ovary is located. The pollen grains (pollinia) are located at its top.

To date, studies of the *AP2*-like genes in orchids are restricted to species belonging to the subfamily Epidendroideae. The *DcruAP2* gene of *Dendrobium crumenatum* is expressed in all floral organs [40], and the twelve *EpAP2*-like genes of *Erycina pusilla* are expressed at different levels in flower and vegetative tissues [41]. The function of the microRNA mir172 in the cleavage of *AP2*-like mRNA was also demonstrated in *E. pusilla* and *Phalaenopsis aphrodite* (Epidendroideae) [41], [42].

In this study we report the isolation, genomic characterization and expression analysis of the *AP2*-like gene *OitaAP2* in the orchid *Orchis italica* (Orchidoideae) and demonstrate that it is subjected to alternative splicing. In addition, we verified the presence of cleaved products generated by the interaction of miR172 with its specific target site within the *OitaAP2* mRNA, analyzed the expression pattern of miR172 within the floral tissues of *O. italica* and compared it to that of the *OitaAP2* gene.

## Materials and Methods

### Isolation of the *OitaAP2* cDNA

Total RNA was extracted from floral buds of *O. italica* using the TRIzol Reagent (Ambion). After DNase treatment (Ambion), RNA was quantified using a Nanodrop 2000c spectrophotometer (ThermoScientific) and reverse-transcribed (1 µg) using the Advantage RT-PCR kit (Clontech) and oligo dT primer. The degenerate forward primer AP2F2 (Table 1), which matches a region of the nucleotide sequence encoding the conserved AP2 domain, and the oligo dT primer were used to amplify 1 µl of cDNA using the LongAmp Taq PCR Kit (New England Biolabs), following the manufacturer instructions. The amplification products were cloned into the pGEM-T Easy vector (Promega), and several clones were sequenced using the plasmid primers T7 and SP6 and an ABI 310 Automated Sequencer (Applied Biosystems). Based on the obtained nucleotide sequences, two specific reverse primers (AP2R1 and AP2R2, Table 1) that anneal downstream of the region encoding the AP2 domains were designed to amplify the 5′-coding and UTR terminus of the putative *AP2*-like cDNA using the FirstChoice RLM-RACE Kit (Ambion). The amplification product was cloned and sequenced as described above. The nucleotide sequences of the 5′- and 3′- regions of the putative *AP2*-like gene were overlapped using the software BIOEDIT [43], and the resulting full length sequence (called *OitaAP2*) was used to perform BLAST analysis.

**Table:** Table 1. Nucleotide sequences of the primers used to amplify the cDNA and the genomic DNA of the *OitaAP2* locus of *Orchis italica.*

Name	Direction	Sequence (5′-3′)	Position
AP2F8	F	ATGGTGCTAGATCTCAACGTGTCAT	1–25
AP2F4new	F	CATCAAACTCCTCTGTTCTCAATG	89–112
AP2F1	F	GATGGGARTCKCAYATYTGGGA	509–530
AP2F6	F	GGAAGAATTTGTGCATATTCTTCGG	690–714
AP2F5	F	CTTCACAAATGTGGGCGGTG	766–785
AP2R5	R	CACCGCCCACATTTGTGAAG	785–766
AP2F2	F	TGGGARGCTMGNATGGGNCARTT	780–806
AP2RealF_iso	F	CAAGAAATTGAAGGAAAGGGCCATGG	1089–1101; 1207–1219
AP2F4old	F	GAGCATCCTCATGTTTGGGGCAG	1153–1175
AP2R4old	R	CTGCCCCAAACATGAGGATGCTC	1175–1153
AP2RealF	F	TGTGTACCCCGGATTATTTCCT	1176–1197
AP2R2	R	AGGAAATAATCCGGGGTACACA	1197–1176
AP2R1	R	TTTCTGGGGCCAAGTGGTCATGGT	1260–1237
APF3	F	TGCAGCATCATCAGGATTC	1296–1314
APR3	R	GAATCCTGATGATGCTGCA	1314–1296
AP2R9	R	TCAGCTCTGAAAGAAGTGATGACG	1431–1407
AP2R4	R	CCTCTGGCTTCATTTGATATTGAG	1558–1535 (5′-UTR)

The primer positions are numbered starting from the first base of the ATG start codon (position 1) of the *OitaAP2* cDNA.

Based on the BLASTX results, the amino acid sequences of AP2-like proteins were downloaded and aligned to the virtual translation of the OitaAP2 cDNA using the CLUSTAL OMEGA online tool. The resulting alignment was manually adjusted and used to construct the Maximum Likelihood tree using the software MEGA5 [44].

Several primers pairs were designed and used in PCR amplifications of the *OitaAP2* cDNA to verify the existence of alternative splicing events (Table 1). All the obtained amplification fragments were cloned and sequenced as described above. The alternatively spliced form of the *OitaAP2* gene was named *OitaAP2_ISO*.

### Amplification of genomic DNA

**Figure 1:**
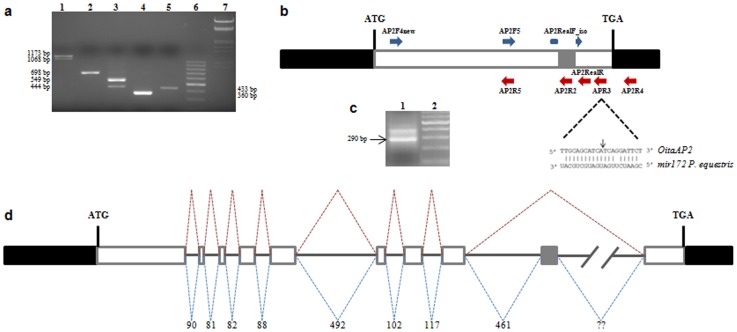
Alternative splicing, mir172 target site and genomic organization of the *OitaAP2* gene of *O. italica*. (**a**) Agarose gel electrophoresis of the PCR amplification products of the *OitaAP2* cDNA with different primer pairs: lane 1, AP2F4new/AP2RealR; lane 2, AP2F4new/AP2R5; lane 3, AP2F5/APR3; lane 4, AP2RealF_iso/AP2R4; lane 5, AP2F5/AP2R2; lane 6, 100 bp DNA ladder (Thermo Scientific); lane 7, DNA Marker III (Thermo Scientific). (**b**) Schematic representation of the *OitaAP2* cDNA with the relative positions of the primers used in the PCR amplifications. Black boxes represent the 5′- and 3′-UTRs; white boxes represent the exons; gray box represents the alternatively spliced region; dotted lines highlight the region corresponding to the miR172 target site with the black arrow indicating the cleavage point. (**c**) Agarose gel electrophoresis of the PCR amplification product of the *OitaAP2* cDNA fragment cleaved by miR172 (lane 1); lane 2, 100 bp DNA ladder (Thermo Scientific). (**d**) Schematic representation of the genomic organization of the *OitaAP2* gene. Black boxes represent the 5′- and 3′-UTRs; white boxes represent the exons; gray box represents the alternatively spliced exon; continuous lines represent introns; dotted lines indicate the two alternative splicing events; numbers indicate the length of introns (bp); question marks indicate the unknown size of intron 9.

Genomic DNA was extracted from *O. italica* leaf tissue [45] and used as a template in amplification reactions using several primer pairs (Table 1). PCR amplifications were performed using the LongAmp Taq PCR Kit (New England Biolabs) following the manufacturer instructions. The genomic region that includes intron 9 was particularly difficult to amplify. Different approaches were used, producing partially successful results that allowed us to map the position of intron 9. In brief, starting from 200 ng of genomic DNA of *O. italica*, a single strand elongation reaction was conducted using the AP2R4 primer (Table 1, Figure 1b). A poly-C tail was added to the 3′-OH end of the obtained single stranded DNA using Terminal deoxynucleotidyl transferase (Invitrogen). The reaction product was PCR-amplified using the forward primer 5′-GGCCACGCGTCGACTAGTACGGGIIGGGIIGGGIIG-3′ and the nested *OitaAP2*-specific reverse primer AP2R9 (Table 1). All amplification products were cloned and sequenced as described above. The obtained nucleotide sequences were aligned and compared to the sequence of the *OitaAP2* and *OitaAP2_ISO* cDNAs.

### Identification of the miR172-cleaved *OitaAP2* mRNA

The 5′-end of the *OitaAP2* mRNA cleavage product produced by miR172 was determined by a modified 5′-RACE [46] using the RLM-RACE GeneRace kit (Invitrogen). In brief, starting from 500 ng of total RNA extracted from early column tissue, the 5′ adaptor was ligated to the 5′-terminus of the RNA without any enzymatic treatment to remove the 5′ cap. After reverse transcription, cDNA was amplified with the GeneRace 5′ Primer and the *OitaAP2* gene-specific reverse primer AP2R4 (Table 1) that anneals within the 3′-UTR, downstream of the miR172 cleavage site. The amplification product was cloned and sequenced as described above.

### Expression analysis of *OitaAP2* and miR172

Total RNA was extracted as described above from outer and inner tepal, lip and column dissected from inflorescence of *O. italica* at two different stages: before anthesis (early, ∼9 mm diameter size) and after anthesis (late). In the early stage (the bud stage), cell division and flower organ formation are already completed; however, cell elongation is still occurring. Total RNA was extracted from unpollinated ovary tissue and, after manual fertilization, from ovary tissue collected 3, 7 and 10 days after pollination (dap). Before pollination, the ovules of *O. italica* are immature, and the megaspore mother cell is undergoing the first meiotic division. At 3 days after pollination, the female gametophyte is completely developed; at 7 days after pollination, fertilization has occurred and the seeds are in early developmental stages; at 10 days after pollination, the seeds are almost mature with seed coats completely developed (Barone Lumaga, personal communication). In addition, total RNA was extracted from root, stem and leaf tissue. After DNase treatment, RNA was quantified as described above.

For expression analysis of the *OitaAP2* gene, 350 ng of total RNA from each tissue was reverse transcribed as described above. The isoform-specific primer pairs AP2RealF/APR3 and AP2RealF_iso/APR3 (Table 1) that selectively amplify a fragment of the two alternatively spliced *OitaAP2* mRNAs were used in the Real Time RT-PCR experiments.

For expression analysis of the microRNA miR172, the Poly(T) Adaptor RT-PCR method was used [47]. Starting from 350 ng of total RNA from each tissue, a poly-T adaptor was ligated to the 3′-terminus of the miRNAs and subsequently, reverse transcription was performed. The forward primer specific for miR172 was designed based on the nucleotide sequence of miR172 in *P. aphrodite* [42] and was used in combination with the poly-T adaptor reverse primer during the Real Time PCR experiments [47]. The Poly(T) Adaptor RT-PCR product of several samples was cloned and sequenced, confirming the specificity of the amplification reaction.

Real time PCR experiments were performed on 30 ng of first strand cDNA from each tissue using the actin *OitaAct* as an endogenous control gene (GenBank accession number AB630020) using the conditions previously described [38]. Reactions were run in technical and biological triplicates. The Real Time PCR Miner online tool [48] was used to calculate PCR efficiency and optimal threshold cycle (C_T_) for each well. The mean relative expression ratio (rER) and standard deviation of the *OitaAP2* gene and of the miR172 microRNA in the different tissues were calculated within each technical triplicate using *OitaAct* as the endogenous control gene and leaf cDNA as the reference sample [49]. Subsequently, the mean rER relative to each tissue of each biological replicate was averaged and standard deviation was calculated. Differences in the relative expression levels of the *OitaAP2* isoforms and mir172 between and/or among the different tissues were assessed by the two-tailed t test and ANOVA followed by the Tukey HSD post-hoc test, respectively.

### In situ hybridization

Inflorescences before anthesis were fixed in 4% (v/v) paraformaldehyde, 0.5% (v/v) glutaraldehyde, 0.1% Triton X-100 and 4% dimethylsulfoxide in phosphate-saline buffer 1X for 16 h at 4°C [50] and then dehydrated through an ethanol series, paraffin embedded and sectioned at 7 µm.

Two different digoxigenin-labeled sense and antisense RNA probes, one corresponding to exon 9 and specific for the *OitaAP2* isoform and the other including the 3′-end of exon 8 followed by the 5′-end of exon 10 and specific for the *OitaAP2_ISO* isoform, were synthesized using the T7 and SP6 RNA polymerases and the DIG RNA Labeling kit (Roche). Hybridization and immunological detection of the signals with alkaline phosphatase was performed using the DIG Nucleic Acid Detection kit (Roche) following the manufacturer's instructions.

## Results and Discussion

### The *OitaAP2* cDNA: alternative splicing

The isolation of *OitaAP2* cDNA was performed using first strand cDNA from the inflorescence of *O. italica* as a template in PCR reactions conducted in the presence of a degenerate forward primer that anneals within the region encoding the AP2 domain 1 and the poly-T reverse primer. This reaction produced two different amplification fragments of ∼1200 and 1100 bp that were cloned and sequenced. Alignment of the two sequenced fragments revealed their full nucleotide identity, excluding a 105 bp region that was absent in the shorter fragment. BLAST analysis showed that both fragments encode a partial AP2-like protein. Based on these preliminary results, specific reverse primers were designed to obtain the 5′-terminus of these cDNAs, and, subsequently, a number of forward and reverse specific primers were designed (Table 1) to verify whether the observed fragments could be the product of an alternative splicing event. PCR amplifications of the cDNA from *O. italica* inflorescence were conducted with different primer pairs. When the forward primer AP2F4new (which anneals 88 bp downstream of the ATG start codon) was used in combination with the AP2RealR primer (which anneals 191 bp upstream of the TGA stop codon), two amplification fragments (1173 and 1068 bp) were produced (Figure 1a, lane 1), whereas a single fragment (698 bp) was obtained when the AP2F4new primer was used in combination with the AP2R5 reverse primer (which anneals within the region encoding the AP2 domain 2) (Figure 1a, lane 2). Two amplification fragments (549 and 444 bp) were produced when the forward primer AP2F5 (reverse and complement of AP2R5) was used in combination with the APR3 reverse primer (which anneals ∼60 bp downstream of AP2RealR) (Figure 1a, lane 3). A single amplification fragment (360 bp) was obtained when the primer AP2RealF_iso, which anneals 13 bp upstream and 13 bp downstream of the region missing in the shortest fragment, was used in combination with AP2R4 (which anneals within the 3′-UTR) (Figure 1a, lane 4). A single amplification fragment (433 bp) was obtained when the forward primer AP2F5 was used in combination with the AP2R2 primer (that anneals within the region absent in the shortest fragment) (Figure 1a, lane 5). The relative primer positions are shown in Figure 1b.

These results might be explained by a number of different phenomena (e.g. gene duplication, transposition events, etc). However, all the evidences strongly suggest the existence of an alternative splicing event resulting in two isoforms of different sizes named *OitaAP2* and *OitaAP2_ISO*. The deletion of 105 bp, which differentiates the two isoforms, is in frame with the main ORF. The entire cDNA sequence of the *OitaAP2* cDNA of *O. italica* is 2264 bp (GenBank accession number KF152921), whereas the size of the *OitaAP2_ISO* cDNA is 2159 bp (accession number KF152922). Both *OitaAP2* and *OitaAP2*_*ISO* include identical 5′- and 3′-UTRs of 560 and 273 bp, respectively.

**Figure 2:**
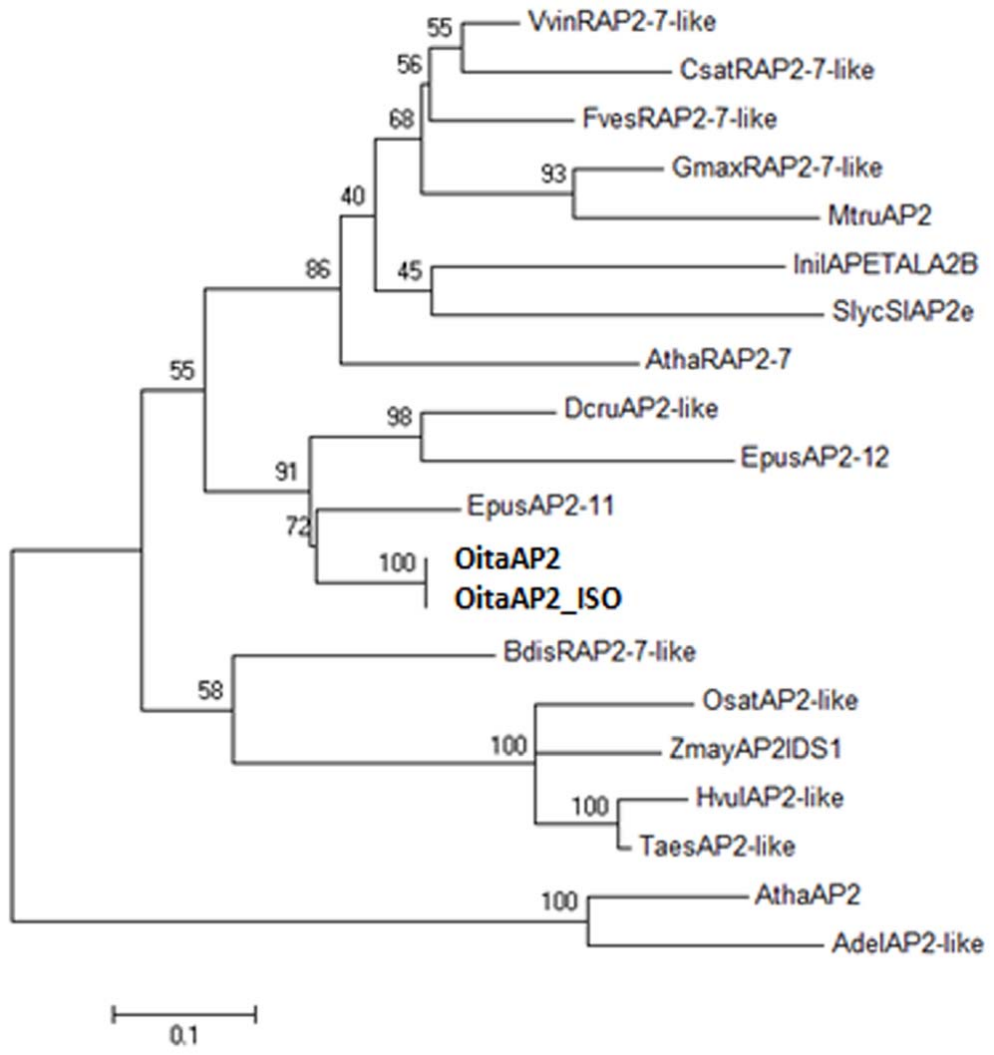
Maximum likelihood tree constructed on the alignment of the AP2-like amino acid sequences belonging to both AP2 and RAP2 groups. Numbers indicate the bootstrap percentages (on 1000 replicates). The abbreviations used (those obtained in the present study are in bold) are in agreement with the GenBank definitions. *Actinidia deliciosa* AdelAP2-like (AER60526); *Arabidopsis thaliana* AthaRAP2-7 and AthaAP2 (NP_001189625 and NP_195410, respectively); *Brachypodium distachyon* BdisRAP2-7-like (XP_003569031); *Cucumis sativus* CsatRAP2-7-like (XP_004148250); *Dendrobium crumenatum* DcruAP2-like (AAZ95247); *Erycina pusilla* EpAP2-11 and EpAP2-12 (AGI62047 and AGI62048, respectively); *Fragaria vesca* FvesRAP2-7-like (XP_004295997); *Glycine max* GmaxRAP2-7-like (XP_003542008); *Hordeum vulgare* HvulAP2-like (AAL50205); *Ipomea nil* InilAPETALA2B (BAD36744); *Medicago truncatula* MtruAP2 (XP_003606515); *Orchis italica* OitaAP2 and OitaAP2_ISO (KF152921 and KF152922, respectively); *Oryza sativa* OsatAP2-like (AAO65862); *Solanum lycopersicum* SlycSlAP2e (NP_001233891); *Triticum aestivum* TaesAP2-like (AAU88192); *Vitis vinifera* VvinRAP2-7-like (XP_002284749); *Zea mays* ZmayAP2IDS1 (NP_001104904).

BLAST analysis revealed the highest similarity with the *AP2*-like loci *EpAP2-11* of the orchid *Erycina pusilla* (74% nucleotide and 68% amino acid identity) and *DcruAP2* of *Dendrobium crumenatum* (72% nucleotide and 60% amino acid identity), followed by the *RAP2-7*-like genes of *Glycine max*, *Vitis vinifera* and other species. The amino acid alignment of the virtually translated sequence of the *OitaAP2* (476 residues) and *OitaAP2_ISO* (441 residues) cDNAs with the AP2-like sequences of different species retrieved from GenBank shows the presence of the two conserved AP2 domains (AP2R1 and AP2R2). In addition, upstream of the AP2 domains are the canonical Motifs 1 and 2 and a nuclear localization signal. A short conserved amino acid stretch located at the C-terminus corresponds to the target site of the microRNA miR172 on the *AP2* mRNA (data not shown). The maximum likelihood (ML) tree shows that the OitaAP2 and OitaAP2_ISO sequences are positioned within the RAP2 group of the AP2-like transcription factors, strictly related to the sequences of the other orchid species (Figure 2). All these features indicate that the *OitaAP2* locus of *O. italica* is member of the *AP2-*like gene subfamily, where it seems to be orthologous to RAP2-7.

Both the *OitaAP2* and *OitaAP2_ISO* cDNAs contain the conserved 21 bp sequence that represents the target site of miR172 on the mRNA. In *Arabidopsis*, miR172 represents a negative post-transcriptional regulator of *AP2*; it cleaves the *AP2* mRNA and acts predominantly by translational inhibition [14], [15], [51]. This regulatory mechanism seems to be conserved among plant species [52], [53], [54]. Using a modified 5′RACE reaction [46], the miR172 5′-end cleavage product of the *OitaAP2/OitaAP2_ISO* mRNA was successfully amplified (Figure 1c). Cloning and sequencing of this fragment (∼290 bp) revealed the position of the cleavage site within the miR172 target site (Figure 1b). The nucleotide sequence of the other fragment (∼390 bp) detectable in Figure 1c showed it is a PCR artifact. According to the results obtained in *P. aphrodite* and *E. pusilla* [41], [42], this finding demonstrates that in orchids, the regulatory mechanism that determines the translational repression of *OitaAP2* through miR172 is conserved.

### Genomic structure of the *OitaAP2* locus

Based on the differential splicing observed for the *OitaAP2* mRNA, it was necessary to characterize the genomic organization of the *OitaAP2* locus of *O. italica*. To evaluate and compare the structure of the *OitaAP2* gene with that of known *AP2*-like genes, the *OitaAP2* locus was amplified from genomic DNA using multiple primer pairs. Sequence comparison of the *OitaAP2* and *OitaAP2_ISO* cDNAs with the genomic sequence of the *OitaAP2* locus (accession number KF152923) revealed the presence of 10 exons and 9 introns (Figure 1d).

This gene structure appears conserved for the *AP2* genes of *Arabidopsis*, grapevine [55] and apple [56], all of which constitute 10 exons and 9 introns. The twelve *AP2*-like genes of the orchid *E. pusilla* show an intron number ranging from 7 to 11; however, the specific structure of the *EpAP2-11* gene, the putative ortholog of *OitaAP2*, is not reported [41]. The size of the exons and the position of the introns are quite conserved among the *AP2*-like genes. All the introns of the *OitaAP2* gene have a relatively small size (ranging from 81 to 492 bp), with the only exception possibly represented by the intron 9. Numerous attempts to amplify this intron were unsuccessful; however, the only approach that resulted in a positive result allowed us to map the position and obtain a partial sequence of intron 9 (∼340 bp). Two hypotheses could explain the difficulties in PCR amplification of this genomic region: the great size of intron 9 and/or its base composition, which might inhibit the polymerase activity. As the amplification condition tested allowed us to amplify large introns (up to 16,000 bp) in other orchid genes (e.g., *OitaAG* and *OitaSTK*) [39] and the single strand DNA extension was interrupted a few hundred nucleotides upstream of the 3′-end of intron 9, the hypothesis of a complex base composition seems more likely than that of an intron size greater than 16,000 bp.

All the introns identified contain the canonical 5′GT and 3′AT, with the only exception being intron 4, which presents a non-canonical 5′GC donor splicing site. CENSOR analysis of the intron sequences showed traces of mobile elements belonging to class I and II transposable elements within the longest intron 5 (Chapaev-16_HM), intron 8 (Copia-10_TC-I) and part of the sequence of intron 9 (CR1-23_CQ) of *OitaAP2*. This result further confirms the abundance of mobile elements within orchid genomes, as detected for the *OitaAG* and *OitaSTK* genes [39].

**Figure 3:**
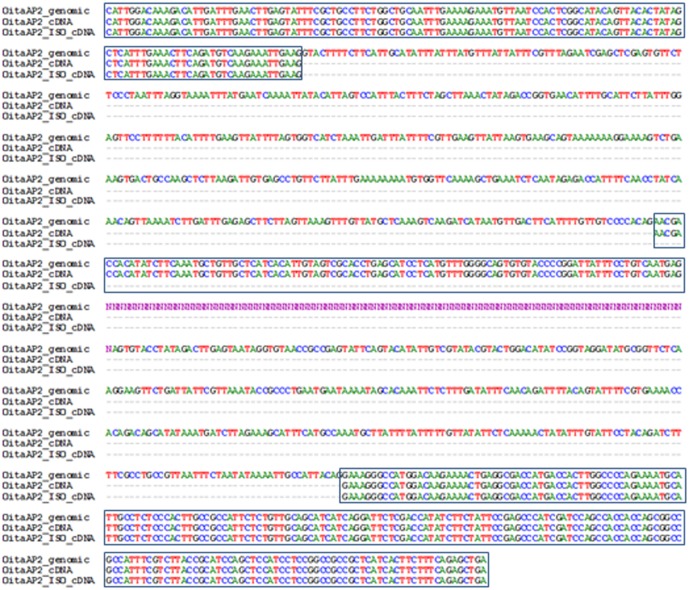
Region of the *OitaAP2* locus of *O. italica* involved in the alternative splicing. Nucleotide alignment spans from exon 8 to exon 10 and includes intron sequences. Boxes indicate exons; Ns indicate unknown nucleotides of intron 9.

Nucleotide comparison of the genomic sequence of the *OitaAP2* locus with the *OitaAP2* and *OitaAP2_ISO* cDNA reveals that the isoform *OitaAP2_ISO* is produced through differential splicing of the region spanning intron 8 to intron 9, with skipping of exon 9 (105 bp) that preserves the correct reading frame (Figure 3). Alternative splicing of the *AP2*-like genes was reported in *Arabidopsis* within the ANT group [41] and more recently in kiwifruit within the AP2 group [54]. To date, alternative splicing of the *RAP2*-like genes has not been described. Similarly to *O. italica*, the region involved in the alternative splicing of the *AdAP2* locus of kiwifruit spans from intron 8 to 9 and includes an unusually large intron. However, the alternative kiwifruit isoform *AdAP2Δ*, whose functional significance is unknown, retains an additional fragment (named exon 9a) that alters the main reading frame and introduces a stop codon and a putative polyadenylation site, excluding the miR172 target site from the transcript.

### Expression pattern of the *OitaAP2* isoforms

**Figure 4:**
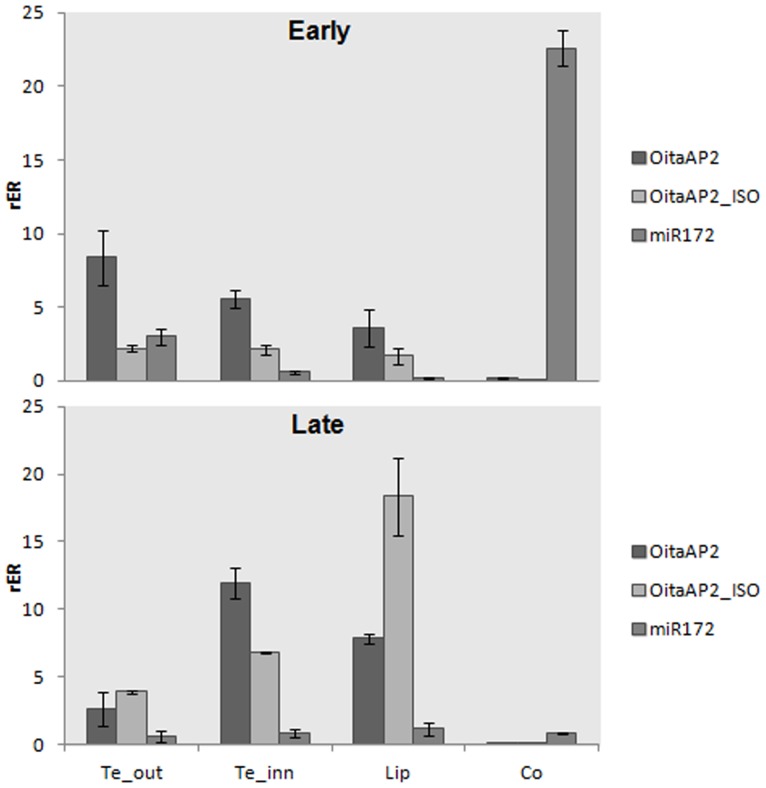
Relative expression ratio (rER) of *OitaAP2*, *OitaAP2_ISO* and mir172 in different tissues of *O. italica* at early and late stages. Te_out, outer tepal; Te_inn, inner tepal; Co, column. Bars represent standard deviation of the biological replicates.

**Figure 5:**
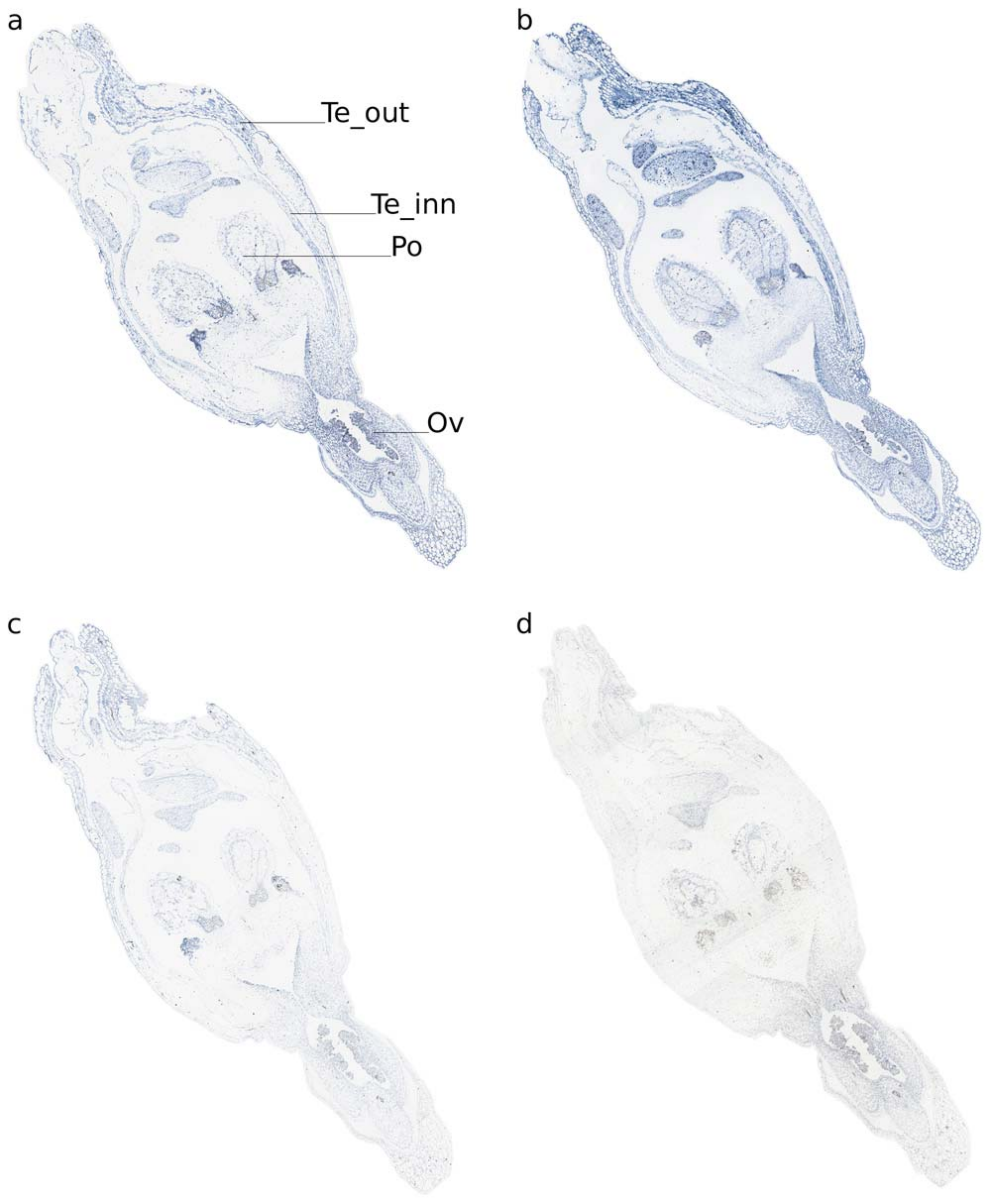
RNA *in situ* hybridization of *OitaAP2* and *OitaAP2_ISO* in inflorescence tissue of *O. italica*. Sections were hybridized with *OitaAP2* and *OitaAP2_ISO* antisense (**a**, **b**) and sense (**c**, **d**) probes. Te_out, outer tepal; Te_inn, inner tepal; Co, column; Ov, ovary; Po, pollinia.

**Figure 6:**
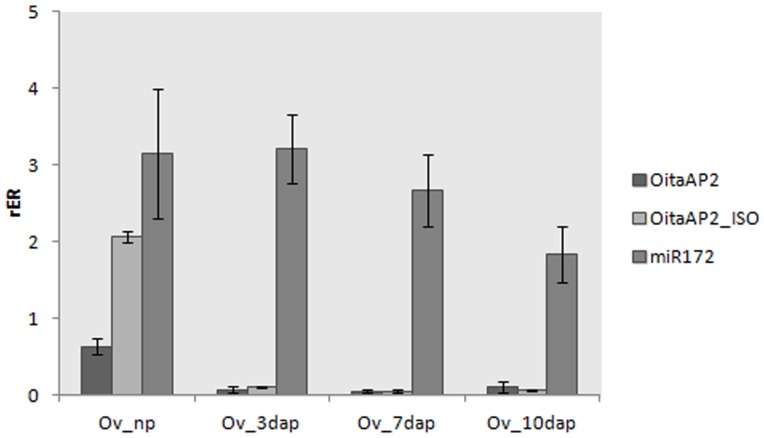
Relative expression ratio (rER) of *OitaAP2*, *OitaAP2_ISO* and mir172 in ovary tissue of *O. italica* before and after pollination. Ov_np, unpollinated ovary; Ov_3dap, Ov_7dap, and Ov_10dap, ovary 3, 7 and 10 days after pollination, respectively. Bars represent standard deviation of the biological replicates.

**Figure 7:**
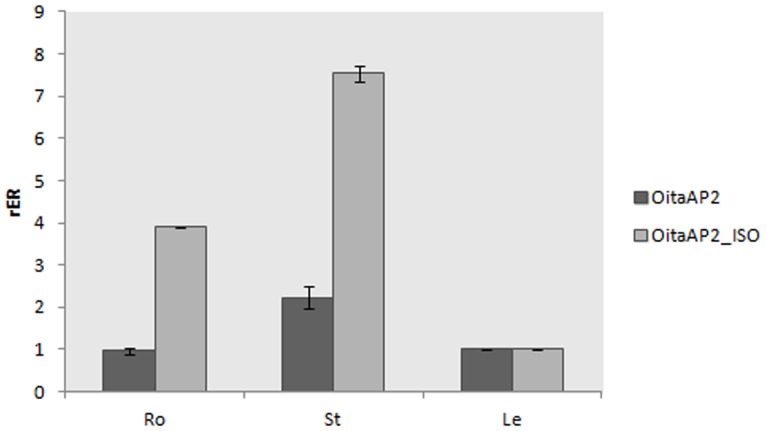
Relative expression ratio (rER) of *OitaAP2* and *OitaAP2_ISO* in vegetative tissues of *O. italica*. Ro, root; St, stem; Le, leaf. Bars represent standard deviation of the biological replicates.

Real-time RT-PCR experiments were performed to reveal the expression pattern of the *OitaAP2* gene in *O. italica*. Isoform-specific reverse primers were used to distinguish between the expression profile of *OitaAP2* and *OitaAP2_ISO* in floral tissues from early and late inflorescence. The melting curve plots demonstrated the effectiveness of the primers used. The mean PCR efficiency for each gene (the target isoforms *OitaAP2* and *OitaAP2_ISO* and the endogenous control *OitaAct*) showed comparable values (data not shown). Both isoforms are ubiquitously expressed in the perianth in early and late inflorescence and are absent in the column (Figure 4). Specifically, in the early inflorescence *OitaAP2* is more highly expressed in the outer and inner tepals than in the lip, whereas in the same tissues *OitaAP2_ISO* is expressed at levels always significantly lower than those detected for *OitaAP2*. In late inflorescence, the expression level of *OitaAP2* decreases in the outer tepals and increases in the inner tepals and lip, whereas the amount of *OitaAP2_ISO* mRNA increases in outer and inner tepals and, more strongly, in the lip (Figure 4). RNA *in situ* hybridization confirms the expression of the *OitaAP2* and presumably of the *OitaAP2_ISO* isoform in all the organs of the perianth (Figure 5). The relative expression ratio of *OitaAP2* and *OitaAP2_ISO* was evaluated also in ovary tissue before and after pollination (at 3, 7 and 10 dap) (Figure 6). Both isoforms are expressed only in ovary before pollination, where the *OitaAP2_ISO* is significantly more abundant than *OitaAP2*. RNA *in situ* hybridization confirms the expression of the *OitaAP2* and presumably of the *OitaAP2_ISO* isoform in the ovary tissue before pollination (Figure 5). Relative expression analysis of *OitaAP2* and *OitaAP2_ISO* in vegetative tissues revealed very low amounts of *OitaAP2* mRNA, with a slight increase in stem tissue. In contrast, the expression of the *OitaAP2_ISO* isoform is significantly higher in root and stem tissue (Figure 7).

In orchids, the *DcruAP2* gene of *D. crumenatum* is expressed in all floral organs and leaves without differential splicing [40], and ML phylogenetic analysis revealed that this gene is orthologous to the *EpAP2-12* gene of *E. pusilla*, whereas the *OitaAP2* gene of *O. italica* is orthologous to the *EpAP2-11* gene (Figure 2), homologous to the *RAP2.7* gene of *Arabidopsis* and expressed both in floral and vegetative tissues [41]. Studies conducted in *Arabidopsis*, rice and grapevine confirmed the expression of the *RAP2-7*-like genes in floral and vegetative tissues [2], [34], [35]. However, differential splicing has never been described for these genes. In *O. italica*, the expression pattern of both isoforms, *OitaAP2* and *OitaAP2_ISO*, is restricted to the outer and inner tepals and lip and is absent in the fused reproductive organs (column). The different expression level of the two isoforms, in particular in the lip from the late inflorescence, where the highest expression of the *OitaAP2_ISO* isoform is detected, might reflect a possible functional partition of the two isoforms in the development and maintenance of the perianth organs. The expression profile detected in the ovary before pollination and in vegetative tissues seems to confirm non-redundant functions of *OitaAP2* and *OitaAP2_ISO*, with the latter specifically involved in ovary formation (but not in post-pollination processes) and in root- and stem-specific functions. However, further studies are needed to confirm this hypothesis and to verify the presence of two *AP2* isoforms in other orchid species.

### Comparative expression analysis of the *OitaAP2* isoforms and miR172

*AP2*-like genes are negatively regulated by the microRNA miR172. To evaluate the relationship existing between *OitaAP2* and mir172 in *O. italica*, their relative expression patterns in different floral tissues was compared (Figure 4).

In the tissues from early inflorescence, the expression profile of miR172 was clearly complementary to that observed for the *OitaAP2* isoforms. In particular, miR172 appears to be expressed mainly in the column from early inflorescence and is almost absent in the other tissues. This pattern fully agrees with the repressive action of mir172 on the *OitaAP2* isoforms. Surprisingly, in the floral tissues from late inflorescence, miR172 is expressed at very low levels in all tissues, including the column. This behavior suggests that in *O. italica* the inhibitory role of miR172 on the *OitaAP2* transcripts is exerted until the stages preceding the anthesis, whereas the absence of *OitaAP2* mRNA in the column after anthesis might be related to different regulatory mechanisms.

In the ovary tissue, miR172 is expressed from pre-pollinated ovary to 10 days after pollination, as observed in *Arabidopsis* [27], showing its possible involvement in ovary development. However, in the ovary tissue of *O. italica*, the miR172 expression pattern is not complementary to that observed for the *OitaAP2* isoforms (Figure 6). These results also suggest that during ovary maturation the inhibition of the *OitaAP2* isoforms might not be directly realized through miR172-mediated cleavage.
